# Effects of Diesel Engine Exhaust Origin Secondary Organic Aerosols on Novel Object Recognition Ability and Maternal Behavior in BALB/C Mice

**DOI:** 10.3390/ijerph111111286

**Published:** 2014-10-30

**Authors:** Tin-Tin Win-Shwe, Yuji Fujitani, Chaw Kyi-Tha-Thu, Akiko Furuyama, Takehiro Michikawa, Shinji Tsukahara, Hiroshi Nitta, Seishiro Hirano

**Affiliations:** 1 Center for Environmental Health Sciences, National Institute for Environmental Studies, 16-2 Onogawa, Tsukuba, Ibaraki 305-8506, Japan; E-Mails: tmichikawa@nies.go.jp (T.M.); nitta@nies.go.jp (H.N.); 2 Center for Environmental Risk Research, National Institute for Environmental Studies, 16-2 Onogawa, Tsukuba, Ibaraki 305-8506, Japan; E-Mails: fujitani.yuji@nies.go.jp (Y.F.); kawagoe@nies.go.jp (A.F.); seishiro@nies.go.jp (S.H.); 3 Division of Life Science, Graduate School of Science and Engineering, Saitama University, 255 Shimo-Okubo, Sakura-ku, Saitama City, Saitama 338-8570, Japan; E-Mails: chawchaw25@gmail.com (C.K.-T.-T.); stsuka@mail.saitama-u.ac.jp (S.T.)

**Keywords:** diesel exhaust, secondary organic aerosol, brain, memory function, maternal behavior, mice

## Abstract

Epidemiological studies have reported an increased risk of cardiopulmonary and lung cancer mortality associated with increasing exposure to air pollution. Ambient particulate matter consists of primary particles emitted directly from diesel engine vehicles and secondary organic aerosols (SOAs) are formed by oxidative reaction of the ultrafine particle components of diesel exhaust (DE) in the atmosphere. However, little is known about the relationship between exposure to SOA and central nervous system functions. Recently, we have reported that an acute single intranasal instillation of SOA may induce inflammatory response in lung, but not in brain of adult mice. To clarify the whole body exposure effects of SOA on central nervous system functions, we first created inhalation chambers for diesel exhaust origin secondary organic aerosols (DE-SOAs) produced by oxidation of diesel exhaust particles caused by adding ozone. Male BALB/c mice were exposed to clean air (control), DE and DE-SOA in inhalation chambers for one or three months (5 h/day, 5 days/week) and were examined for memory function using a novel object recognition test and for memory function-related gene expressions in the hippocampus by real-time RT-PCR. Moreover, female mice exposed to DE-SOA for one month were mated and maternal behaviors and the related gene expressions in the hypothalamus examined. Novel object recognition ability and N-methyl-d-aspartate (NMDA) receptor expression in the hippocampus were affected in male mice exposed to DE-SOA. Furthermore, a tendency to decrease maternal performance and significantly decreased expression levels of estrogen receptor (ER)-α, and oxytocin receptor were found in DE-SOA exposed dams compared with the control. This is the first study of this type and our results suggest that the constituents of DE-SOA may be associated with memory function and maternal performance based on the impaired gene expressions in the hippocampus and hypothalamus, respectively.

## 1. Introduction

Epidemiological studies have indicated that inhalation of elevated levels of particulate matter (PM) is associated with an increase in pulmonary and cardiovascular morbidity and mortality in susceptible populations [1,2,3,4]. Diesel exhaust (DE) is a major source of fine ambient PM in urban environments. Several studies have indicated that DE exposure may affect the central nervous system. In human studies, neurobehavioral impairment was observed in railroad workers exposed to DE [5], and altered electrical signals in the frontal cortex was found in human volunteers exposed to DE [6]. Secondary organic aerosols (SOAs) are formed in the atmosphere by oxidation of products from anthropogenic and biogenic volatile organic compounds [7] and DE is one of the major precursors for SOA formation. Currently, the importance of SOA formation in urban areas is well recognized, not only in the atmosphere but also in indoor environments [8,9]. Recently our research group has shown that an acute single intranasal administration of SOA induces inflammatory responses in the lung by modulating proinflammatory cytokines, transcription factors and inflammatory responsive neurotrophins [10]. In that study, we found that an acute single intranasal instillation of SOA does not affect biomarkers in the brain of normal healthy individuals. However, there are limited reports on the association between exposure to DE-origin SOA and central nervous system functions such as learning and memory and maternal performance. Therefore, we sought to study the effect of subchronic inhalation exposure to SOA on higher brain function. There are very few studies regarding health effects of SOA in animal and humans and this is mainly due to the lack of suitable particle exposure techniques for studies of *in vivo* and *in vitro* toxicity effects of SOA. We first established the whole body SOA exposure chambers and a system for oxidation of diesel exhaust particles (DEP) with ozone at our National Institute for Environmental Studies (Tsukuba, Japan). Using well-developed SOA exposure chambers, we investigated the effects of subchronic SOA exposure on memory function and maternal behavior in mice.

Regarding animal studies, our laboratory has reported that exposure to ultrafine or nanosized particles from DE impaired spatial learning ability, long-term memory, expressions of memory function-related genes, inflammatory biomarkers and neurotransmitter release in the brain of adult mice [11,12,13,14,15]. In addition, it was shown that the short-term exposure to road side pollution affects the expression levels of inflammatory genes, up-regulation in the hippocampus and down-regulation in olfactory bulbs of mice [16]. Long-term exposure to air pollution can trigger blood brain barrier disruption, neuroinflammation and neurodegeneration [17,18]. In human studies, Calderon-Garciduenes and colleagues have reported that children who have prefrontal lesions exposed to air pollution in Mexico City showed cognitive deficits [19,20]. Moreover, some studies have demonstrated an association between air pollution and cognitive impairment in healthy individuals, including adult and elderly women [21,22,23]. Atmospheric aerosols also have an important impact on human health and it is now well established that exposure to ambient aerosols is associated with adverse effects on the respiratory and cardiovascular systems [24,25]. However, the effects of DE-origin SOA (DE-SOA) exposure on central nervous system remain largely unknown. Our understanding of health risk effects of SOA is a priority topic for public health research and this prompted us to do the present study, which purpose is to investigate the effects of DE-SOA on higher brain functions such as memory function and maternal behavior in a mouse model. We hypothesized that the constituent(s) of SOA may reach the brain via the olfactory nerve route or systemic circulation and may then affect the brain by producing oxidative stress and modulating neurological and hormonal biomarkers. This is the first study to report that ultrafine components of SOA may be associated with central nervous system functions.

## 2. Materials and Methods

### 2.1. Animals

Male and female BALB/c mice (4 weeks old) were purchased from Japan SLC Co. (Tokyo, Japan). Five-week-old mice were used in the experiments. Male mice were used for one or three month exposure schedules and allotted to three groups (*n* = 8 for each group): control, DE and DE-SOA-exposed groups. During the exposure period, animals were kept in the respective inhalation chambers. Regarding learning behavioral tests, male mice were kept in a group (*n* = 8) in one cage in each exposure chamber. For maternal behavior experiment, we performed mating of female mice exposed to DE or DE-SOA for one month with same aged male mice without exposure (*n* = 8). Food (a commercial CE-2 diet, CLEA Japan, Inc., Tokyo, Japan) and water were given *ad libitum*. The male mice were housed in the wire cages under controlled environmental conditions (temperature, 22 ± 0.5 °C; humidity, 50 ± 5%; lights on 07:00–19:00). For assessment of maternal behavior, pregnant mice were kept individual cages (eight cages) in each exposure chamber under the same controlled environmental conditions as male mice. The cage for pregnant mice was a special cage (thick saw dust flooring to avoid stress) different from the usual wire cage that we usually use for exposure of pregnant mice in our Institute. This study was approved by the Ethics Committee of the Animal Care and Experimentation Council of the National Institute for Environmental Studies (NIES), Japan.

### 2.2. Generation of Secondary Organic Aerosol (SOA)

Diesel exhaust origin SOA was generated at the National Institute for Environmental Studies, Japan. An 81-diesel engine (J08C; Hino Motors Ltd, Hino, Japan) was used to generate diesel exhaust. Detail of the exposure system is described in Fujitani *et al.* [26]. The engine was operated under steady-state conditions for 5 h per day. In the present study, our diesel engine driving conditions did not simulate any special operating regime as in the real world. The engine operating conditions (2000 rpm engine speed and 0 Nm engine torque) in this study permit suppression of the generation of soot particles of relatively large size as well as the generation of high concentrations of nanoparticles. There are three chambers: a control chamber receiving clean air filtered using a HEPA filter and a charcoal filter (referred to as “clean air”), the diluted exhaust (DE not mixed with O_3_), and DE-SOA which was generated by mixing DE with O_3_ at 0.6 ppm after secondary dilution. The secondary dilution ratios in the DE and DE-SOA chambers were the same, so the particle and gaseous concentrations would be the same as when O_3_ was not added. In fact, though, the concentrations of particles in DE-SOA was higher when O_3_ was mixed and the concentrations of DE and DE-SOA were 97.69 ± 3.6 μg/m^3^ and 113.99 ± 3.1 μg/m^3^, respectively. To quantify real human exposure level (that is 35 μg/m^3^), the concentration of 113.99 μg/m^3^ in our present study was approximately 16.8 μg/m^3^ because the exposure time is 5 h per day for 5 days per week. The increased mass concentration was due to secondary generated particles. The temperature and relative humidity inside each chamber were adjusted to approximately 22 ± 0.5 °C and 50 ± 5%, respectively. The particle characteristics were evaluated from the sample air taken from inside of the exposure chamber (Table 1). In detail, sample air was taken from the breeding space of the inhalation chamber (2.25 m^3^) using stainless steel tubing. The gas concentrations (CO, CO_2_, NO, NO_2_, and SO_2_) were monitored using a gas analyzer (Horiba, Kyoto, Japan) (Table 1). CO and NOx concentrations in both chambers were similar, but NO and NO_2_ are different each other because NO was oxidized to NO_2_ by reaction with O_3_. The particle size distributions were measured using a scanning mobility particle sizer (SMPS 3034; TSI, Shoreview, MN, USA). The sizes of the particles used in the present study were 31.48 ± 0.7 nm for DE and 32.78 ± 0.9 nm for DE-SOA. The particles were collected using a Teflon filter (FP-500; Sumitomo Electric, Osaka, Japan) and a Quartz fiber filter (2500 QAT-UP; Pall, Pine Bush, NY, USA), and the particle mass concentrations were measured using a Teflon filter. The particle weights were measured using an electrical microbalance (UMX 2, Mettler-Toledo, Columbus; OH, USA; readability 0.1 μg) in an air-conditioned chamber (CHAM-1000; Horiba) under constant temperature and relative humidity conditions (21.5 °C, 35%). For the Quartz fiber filter, the quantities of elemental carbon (EC) and organic carbon (OC) were determined using a carbon analyzer (Desert Research Institute, NV, USA). EC to OC ratio in the present study were 0.17 ± 0.09 for the control chamber, 0.38 ± 0.02 for DE-SOA chamber and 0.37 ± 0.01 for the DE exposure chamber. An analysis of the particle composition (DE and DE-SOA) showed that the percentage of OC relative to the total carbon in diluted exhaust was about 60% and the DE and DE-SOA had nearly the same carbon composition.

### 2.3. Experimental Schedule

Experiments for long-term memory function: the male mice were allocated into three different groups (*n* = 8 per group) as follows: (1) mice exposed to clean filtered air; (2) mice exposed to DE and (3) mice exposed to DE-SOA. Mice were exposed to clean air, DE or DE-SOA in the whole-body exposure chamber (Shibata) for 5 h per day (from 22:00 to 03:00) on 5 days of the week for one or three months. On the day after the final exposure, the learning ability of each mouse was examined using a novel object recognition test. Twenty-four hours after the completion of the novel object recognition test, the mice were sacrificed and six of the eight mice were used for the mRNA analysis and two were used for the immunohistochemical analysis.

Experiments for maternal behavior: We performed mating of female mice exposed to DE or DE-SOA for one month with same aged male mice without exposure. The pregnant mice were allocated into three different groups (*n* = 8 per group) as follows: (1) mice exposed to clean filtered air; (2) mice exposed to DE and (3) mice exposed to DE-SOA. After birth, pups were kept with the dam till postnatal day (PND) 21. On the day of PND5 or 12, maternal behaviors were examined by a video-assisted tracking system (Muromachi Kikai Co. Ltd., Tokyo, Japan). On the day of PND 28, the dams were sacrificed and six of the eight mice were used for the mRNA analysis and two were used for the immunohistochemical analysis.

**Table 1:** Characterization of diesel exhaust particle and gaseous compounds in the chambers.

Chamber	Particles				Temperature	Relative Humidity
	Size (nm)	Particles Number (cm^−3^)		Concentration (µg/m^3^)	(°C)	(%)
Clean air	--	2.90 ± 0.43		15.70 ± 0.63	21.99 ± 0.08	52.99 ± 0.38
DE-SOA	32.78 ± 0.87	3.16 × 10^6^ ± 6.18 × 10^4^		113.99 ± 3.06	22.08 ± 0.10	50.77 ± 0.68
DE	31.48 ± 0.71	3.28× 10^6^ ± 4.52 × 10^4^		97.69 ± 3.60	22.02 ± 0.07	51.35 ± 0.65
Chamber	Gaseous Compounds					
	CO (ppm)	SO_2_ (ppm)	NO*_x_* (ppm)	NO_2_ (ppm)	NO (ppm)	CO_2_ (%)
Clean air	0.33 ± 0.05	0.00 ± 0.00	0.00 ± 0.00	0.00 ± 0.00	0.00 ± 0.00	0.05 ± 0.00
DE-SOA	2.61 ± 0.08	0.00 ± 0.00	1.39 ± 0.02	1.09 ± 0.03	0.29 ± 0.03	0.07 ± 0.00
DE	2.52 ± 0.08	0.01 ± 0.00	1.33 ± 0.03	0.43 ± 0.01	0.90 ± 0.02	0.07 ± 0.00

Data were expressed as mean ± SD; Number of experimental replicates: gaseous compounds = 59; particle size and numbers = 11; mass cincentration = 2.

### 2.4. Novel Object Recognition Test

In the present study, we used a novel object recognition test because we wanted to focus on the effects of DE-SOA exposure on cognitive functions. Another reason is that we already had data from this behavioral test following diesel exhaust particle exposure in mice in our previous studies. We performed a novel object recognition test for four days including the habituation phase (15 min/day for two consecutive days), training phase (10 min for one day), and test phase (5 min for one day) in each mouse after the completion of one or three months of exposure to DE, as described previously [15]. Briefly, in the habituation phase, the mouse was placed into a rectangular cage (50 × 50 × 40 cm) made by acryl Plexiglas for 15 min per day for two days without an object. Then, during the training phase, two identical objects (6 × 7 × 8 cm) were placed near the corners of one wall in the rectangular cage (10 cm from each adjacent wall). The mouse was placed in the center of the cage facing theopposite wall and was allowed to explore both objects for 10 min. Exploration was defined as the mouse positioning its nose toward the object at a distance of less than 2 cm. We did not record the time spent sitting or resting against the object. Twenty-four hours after the training phase, during the test phase, one of the old objects was replaced with a novel object (8 × 9 × 10 cm) and presented to each mouse for 5 min and recorded the object exploration time which was defined as the mouse approaching the object by nose within 2 cm using a video-assisted tracking system (Muromachi Kikai Co. Ltd.). To control the odor cues, the open field arena and the objects were thoroughly cleaned with water, dried, and ventilated for a few minutes between mice. Discrimination between two objects was calculated using a discrimination index (DI), [DI = (novel object exploration time/total exploration time) − (familial object exploration time/total exploration time) × 100], which takes into account individual differences in the total exploration time [27]. We also performed object preference tests using the control, DE and DE-SOA exposed mice. The mice were tested randomly in the three groups and the objects used as novel or familiar were counterbalanced across the treatment groups. The novel object recognition tests were done by an examiner blinded to the exposure conditions.

### 2.5. Maternal Behavior Test

Maternal behaviors were assessed for one hour from 11:00 a.m. to 12:00 a.m. noon by the video assisted computer system. Three pups were used for maternal behavior test and placed at the three corners of cage separately. Then, dam was placed at the fourth corner and maternal behaviors were assessed. The following parameters of maternal behavior were recorded by video assisted computer system at PND 5 or 12: (1) the percentage of dam making a nest, (2) the percentage of dam licking her pups, (3) the percentage of dam crouching over her pups and (4) the percentage of dam retrieving her pups. The maternal behavioral tests were done by an examiner blinded to the exposure conditions.

### 2.6. Quantification of the mRNA Expression Levels

After the completion of the novel object recognition test, six male mice from each group were sacrificed under deep pentobarbital anesthesia and the hippocampi were collected from all the mice. Regarding maternal behavior assessment, on the day of PND 28, eight dams from each group were sacrificed under deep pentobarbital anesthesia and hypothalami from six dams were used for mRNA analyses and the whole brain from two dams were used for immunohistochemical analyses. Hippocampi and hypothalami samples were frozen quickly in liquid nitrogen then stored at −80 °C until the total RNA was extracted. Briefly, total RNA extraction from the hippocampi and hypothalami samples was performed using the BioRobot EZ-1 and EZ-1 RNA tissue mini kits (Qiagen GmbH, Hilden, Germany). Then, the purity of the total RNA was examined, and the quantity was estimated using the ND-1000 NanoDrop RNA Assay protocol (NanoDrop, Wilmington, DE, USA), as described previously [9]. Next, we performed first-strand cDNA synthesis from the total RNA using SuperScript RNase H^−^Reverse Transcriptase II (Invitrogen, Carlsbad, CA, USA), according to the manufacturer’s protocol. Next, we examined the mRNA expressions of 18S, N-methyl d-aspartate receptor subtype 1 (NR1), N-methyl d-aspartate receptor subtype 2A (NR2A), N-methyl d-aspartate receptor subtype 2B (NR2B), estrogen receptor (ER) α and oxytocin (OT) and oxytocin receptor (OTR) usinga quantitative real-time RT-PCR method and the Applied Biosystems (ABI) Prism 7000 Sequence Detection System (Applied Biosystems Inc., Foster City, CA, USA). The tissue 18S rRNA level was used as an internal control. The primer sequences used in the present study (NR1, NM_008169; NR2A, NM_008170; NR2B, NM_008171; ER-α, NM_007956; OT, NM_011025; OTR, NM_001081147) were purchased from Qiagen Sample & Assay Technologies. Data were analyzed using the comparative threshold cycle method. Then, the relative expression levels of memory function-related genes and the related transduction pathway molecule mRNAs were individually normalized to the 18S rRNA content in the respective samples and expressed as mRNA signals per unit of 18S rRNA expression.

### 2.7. Measurement of Plasma 8-Hydroxy-2’Deoxyguanosine (8OHdG) Concentration

The day after the completion of a novel object recognition test, male mice were sacrificed under pentobarbital anesthesia and blood sample were collected (*n* = 6 for each group). Plasma 8OHdG concentration was measured using high sensitive 8OHdG Check ELISA kit (Code #KOG-HS10E) according to the manufacturer’s (Nikken Seil Co., Ltd, Fukuroi, Shizuoka, Japan) directions.

### 2.8. Immunohistochemistry

The brains of dam at the PND 28 were removed from two mice from each of the control and DE or DE-SOA-exposed groups after the animals had been deeply anesthetized with sodium pentobarbital; the brains were then fixed in 10% formalin. The fixed brains were dehydrated using a graded series of ethanol, cleared with xylene, and embedded in paraffin. Coronal paraffin sections were cut at a thickness of 10 μm using a microtome and were mounted on 3-aminopropyltriethoxysilane-coated glass slides. ER-α receptor and OTR were detected immunohistochemically in the hypothalamus.

Briefly, the brain sections were immersed in ethanol followed by 10% H_2_O_2_ for 10 min at room temperature. After rinsing in 0.01-M phosphate buffer saline, the sections were blocked with 2% normal swine serum in PBS for 30 min at room temperature and then reacted with goat polyclonal primary antibody [anti-ER-α (diluted 1:1000; Abcam: ab37438; Tokyo, Japan; anti-OTR (diluted 1:1000; Abcam: ab115664; Tokyo, Japan)] in PBS for 1 h at 37 °C. The sections were reacted with biotinylated donkey anti-goat IgG (1:300 Histofine; Nichirei Bioscience, Tokyo, Japan) in PBS for 1 h at 37 °C before and after rinsing in PBS. The sections were then incubated with peroxidase-tagged streptavidin (1:300, ABC KIT) containing PBS for 1 h at room temperature. After rinsing in PBS, ER-α or OTR immunoreactivity was detected using a Dako DAB Plus Liquid System (Dako Corp, Carpinteria, CA, USA).

### 2.9. Statistical Analysis

All the data were expressed as the mean ± standard error (S.E.). The statistical analysis was performed using the StatMate II statistical analysis system for Microsoft Excel, Version 5.0 (Nankodo Inc., Tokyo, Japan). Paired *t* test was used to analyze the object exploration time between familiar and novel object and DI. Messenger RNA expression data were analyzed using a one-way analysis of variance with a *post-hoc* analysis using the Bonferroni/Dunn method. Maternal behavior was analyzed by Fisher’s exact test (Stata 11 software; Stata Corp., College Station, TX, USA) and parameters were shown % of mice showing maternal behavior during one hour (11:00~12:00) period. Differences were considered significant at *P* < 0.05.

## 3. Results

### 3.1. Body and Organ Weights of Male Mice after Novel Object Recognition Test

To determine the general toxicity of DE or DE-SOA exposure, we measured the body and various organ weights of the male mice after the completion of the novel object recognition test at the time of sampling. In both one or three months exposure, body and organ weights of the exposure groups did not differ from the control group, except for lung and thymus in the mice of DE-SOA one month exposure group (data not shown).

### 3.2. Effect of Diesel Exhaust (DE) or Diesel Exhaust Origin Secondary Organic Aerosol (DE-SOA) Exposure on Novel Object Recognition Test

**Figure 1:**
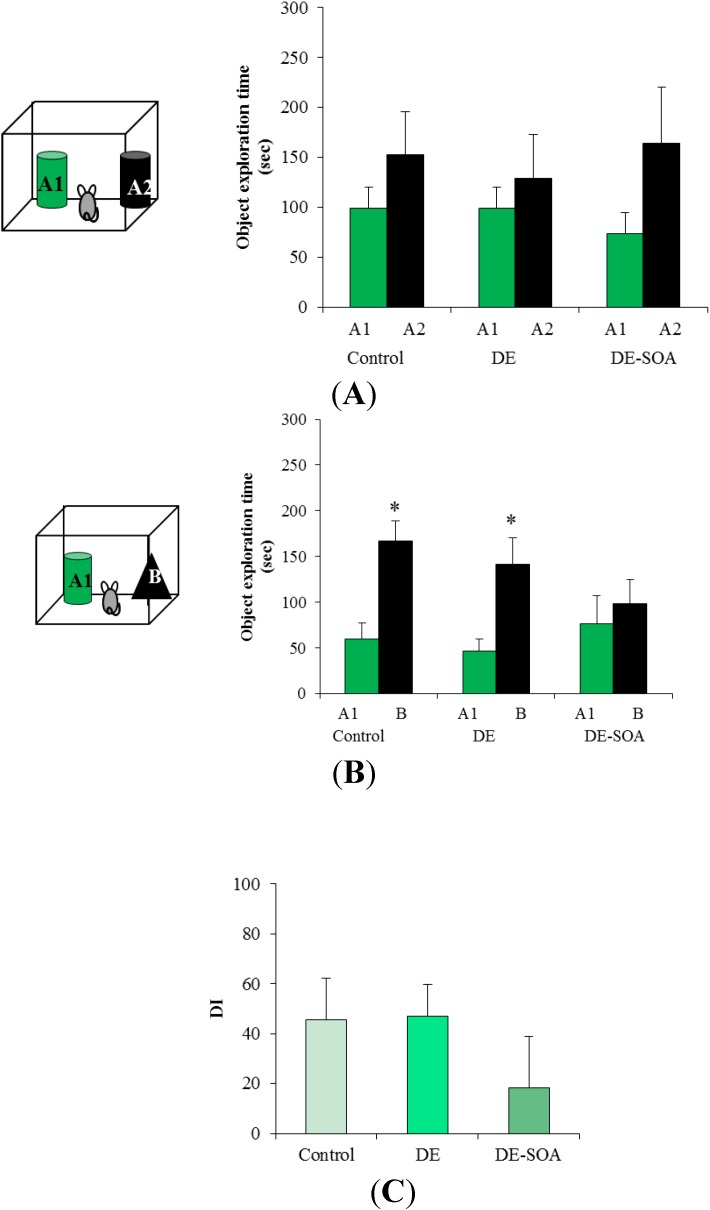
DE-SOA impaired novel object recognition ability after one month exposure. (**A**) Training phase, (**B**) test phase and (**C**) discrimination index in the control, DE and DE-SOA exposed mice. Each bar represents the mean ± SE. A1 and A2 are identical raccoon dog toys and B is bear toy.

Notes: *n* = 8, * *P* < 0.05, paired *t* test, A1 and B in the control and DE exposed groups in test phase.

**Figure 2:**
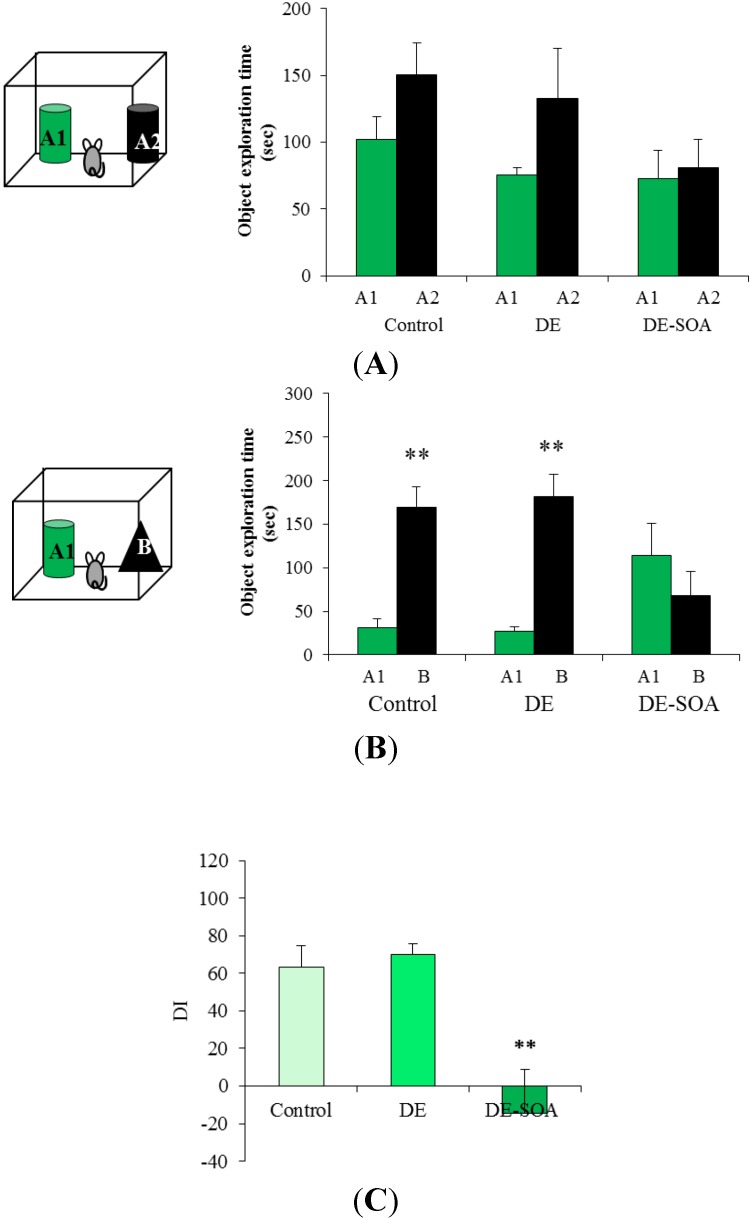
DE-SOA impaired novel object recognition ability after three months exposure. (**A**) Training phase, (**B**) test phase and (**C**) discrimination index in the control, DE and DE-SOA exposed mice. Each bar represents the mean ± SE. A1 and A2 are identical raccoon dog toys and B is bear toy.

Notes: *n* = 8, ** *P* < 0.01, paired *t* test, A1 and B in the control and DE exposed groups in test phase (B); ** *P* < 0.01, paired *t* test, DE-SOA *vs.* control and DE in DI (C).

During the training phase, each mouse was presented to two identical objects for 10 min. In this phase, the object exploration time for both objects was approximately the same for the mice exposed to clean air or NRDE (Figure 1A and Figure 2A).

During the test phase, one of the objects was replaced by a new or novel object, and each mouse was presented to these different objects for 5 min. In this phase, the object exploration time for the novel object was significantly higher in the mice exposed to clean air or DE for one month (*P* < 0.05, Figure 1B) or three months (*P* < 0.01, Figure 2B). We performed training phase for 10 min and test phase for 5 min. From our results, the mice spent 25% of total time (10 min in training phase) at A2 and 50% of total time (5 min in test phase) at B in the control and DE group, but not in DE-SOA group in both one or three month exposure groups.

Discrimination between familiar and novel objects was calculated using a DI. In one month exposure groups, DI was tended to reduce in DE-SOA exposed mice, but not statistically significant (Figure 1C). We found that the DI in DE-SOA exposed mice was significantly reduced compared with that in the clean air or DE-exposed mice in three months exposure groups (*P* < 0.01, Figure 2C). This finding indicates that the mice exposed to DE-SOA for three months could not discriminate a novel object from a familiar object.

### 3.3. Effect of Diesel Exhaust (DE) or Diesel Exhaust Origin Secondary Organic Aerosol (DE-SOA) Exposure on the mRNA Expressions of NMDA Receptor Subunits in the Hippocampus

**Figure 3:**
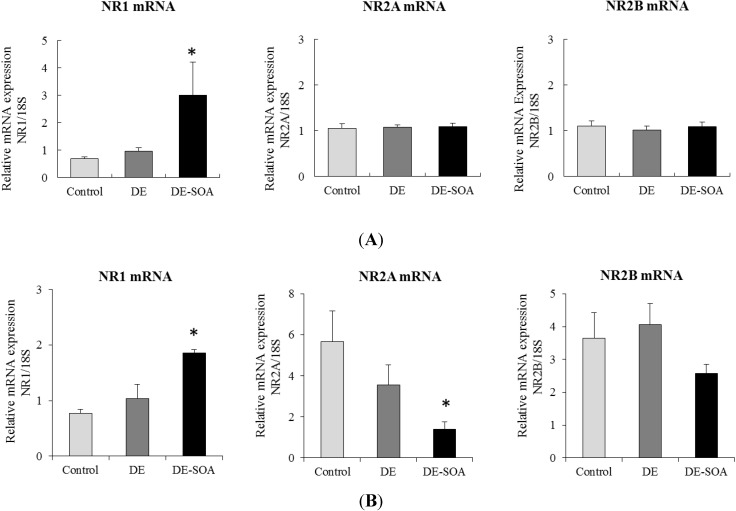
Messenger RNA expression of the NMDA receptor subunits: NR1, NR2A, and NR2B in the hippocampi of mice exposed to clean air, DE, or DE-SOA for (**A**) one month and (**B**) three months. Each bar represents the mean ± SE.

Notes: *n* = 6, *P < 0.05, one way ANOVA, DE-SOA *vs.* control.

To determine the role of NMDA receptor in learning ability, we have also examined the effect DE or DE-SOA exposure on NMDA receptor subunit expression in the mouse hippocampus. In one month exposure groups, we found that the expression levels of NR1, but not NR2A or NR2B, was significantly higher in DE-SOA exposed mice compared to the control and DE-exposed groups (*P* < 0.05, Figure 3A). In three months exposure groups, the expression level of NR1 mRNA was significantly higher in DE-SOA exposed group compared to the other groups, however, NR2A mRNA was remarkably reduced in DE-SOA exposed group compared with the other groups and NR2B mRNA was not different among the groups (*P* < 0.05, Figure 3B).

### 3.4. Effect of Diesel Exhaust (DE) or Diesel Exhaust Origin Secondary Organic Aerosol (DE-SOA) Exposure on Plasma 8OHdG Concentration

8OHdG is commonly used as a marker of DNA oxidative stress [27,28]. 8OHdG level increases in tissue, blood and urine in pathological condition such as cancer, inflammation, diabetes and neurodegeneration. Plasma 8OHdG level was not different between the groups of one month exposure, but it was significantly increased in three months DE-SOA-exposed group compare to the control or DE-exposed group (*P* < 0.05) (data not shown).

### 3.5. Effect of Diesel Exhaust (DE) or Diesel Exhaust Origin Secondary Organic Aerosol (DE-SOA) Exposure on Maternal Behavior

For maternal behavioral assessment, there are several kinds of methods of presentation such as continuous measurement of duration and percentage or number of animals showing maternal performance. In the present study, we focused on showing the overall effects of DE or DE-SOA on maternal behavior because we only used a small sample size, so behavior assessment was performed one hour per day at two time points. Therefore, we considered showing our data as the percentage of mice showing maternal behavior. The mean latency for the the onset of maternal behavior to appear in females was PND 7, with a range from PND 3 to 11 [29]. Therefore, we investigated the maternal behavior for one hour from 11:00 to 12:00 at two time points such as PND 5 and 12. Maternal performance such as nesting and crouching were significantly reduced in DE-SOA exposed dams at PND 5 (Table 2). Some recovery effects were observed in DE-SOA exposed dams at PND 12 (Table 3).

**Table 2:** Percentage of mice showing maternal behavior at PND 5.

Maternal Behavior	Control (%)	DE (%)	DE-SOA (%)
Nesting	100	75	50 *
Licking	100	87.5	87.5
Crouching	87.5	75	12.5 **
Retrieving	12.5	37.5	12.5

Data represent percentage of mice showing maternal behaviors (*n* = 8). * *p* < 0.05 for the difference in the prevalence between control group and DE-SOA group by the fisher’s exact test. ** *p* < 0.05 for the difference in the prevalence between control group and DE-SOA group by the fisher’s exact test.

**Table 3:** Percentage of mice showing maternal behavior at PND 12.

Maternal Behavior	Control (%)	DE (%)	DE-SOA (%)
Nesting	100	62.5	87.5
Licking	100	75	100
Crouching	100	87.5	87.5
Retrieving	0	0	0

Data represent percentage of mice showing maternal behaviors (*n* = 8).

### 3.6. Body and Organ Weights for Maternal Behavior

The body weight of pups, dams and weight of whole brain and some areas of brain (olfactory bulb, hippocampus and hypothalamus) of dam from the exposure groups did not differ from the control group (data not shown). Abnormal maternal data (e.g., delay pregnancy, eating pups) before and just after birth were observed (Table 4).

**Table 4:** Maternal data before and after birth.

Maternal Data	Control (*n* = 8)	DE (*n* = 8)	DE-SOA (*n* = 8)
% of successful pregnancy	100%	100%	100%
% of delayed pregnancy (more than 1 week after mating)	0%	10%	20%
Mother eats pup	0%	12.5%	25%
Range of number of pup	5–6	5–6	4–5
Sex Ratio	male > female	male = female	male = female

Cannibalism (mother eats pups) is an important indicator of maternal behavior and no significant differences between the control and exposure groups were observed by Fisher’s extract test.

### 3.7. Effect of Diesel Exhaust (DE) or Diesel Exhaust Origin Secondary Organic Aerosol (DE-SOA) Exposure on Maternal Behavior-Related Gene Expressions in the Hypothalamus

We have focused on hypothalamus of dam because that area is highly associated with maternal performance. It was reported that the activation of oxytocin receptors and estrogen receptor in the hypothalamus are necessary for maternal behavior in rodents [30,31,32].

**Figure 4:**
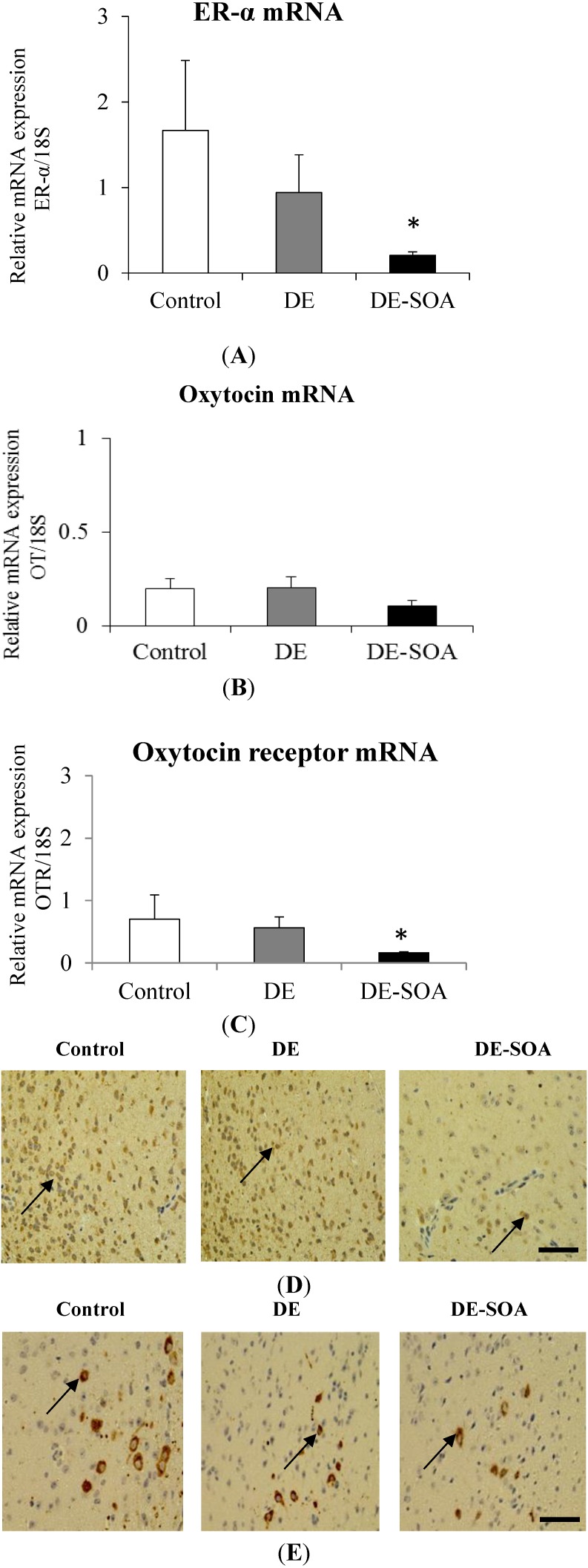
Messenger RNA expression of (**A**) ER-α, (**B**) OT and (**C**) OTR in the hypothalami of mice exposed to clean air, DE, or DE-SOA-exposed dam at the postnatal day 28. Each bar represents the mean ± SE (*n* = 6) (* *P* < 0.05). Representative digital photomicrographs of (**D**) ER-α, and (**E**) OTR immunostained sections of the hypothalamus from clean air, DE, and (**C**) DE-SOA groups. Black arrow indicates the immunoreactive cells (Scale bar = 50 μm).

To investigate the persistent changes of maternal behavior-related gene expressions in the hypothalamus of dam, sampling of dam was done at PND 28 in the present study. We investigated the expression level of ER-α, OT and OTR in the hypothalamus and found that ER-α, and OTR mRNAs in the hypothalamus were significantly reduced in female mice exposed to DE-SOA before pregnancy (*P* < 0.05, Figure 4A,C). In immunohistochemical analysis, immunoreactivity of ER-α and OTR were tended to reduce in DE-SOA exposed mice compared to that of the control mice (Figure 4D,E).

## 4. Discussion

The major findings of the present study are impaired discrimination ability between familiar and novel objects in male mice exposed to DE-SOA for three months and maternal behavior appeared to decrease in female mice exposed to DE-SOA for one month before pregnancy. Moreover, sub-unit specific alteration of learning and memory function-related gene NMDA receptor expression in the hippocampus of adult male mice occurred. Expressions of maternal behavior-related genes such as ER and OTR mRNA were significantly decreased and their immunoreactivity appeared to decrease in hypothalamus of dams exposed to DE-SOA before mating. This is the first study to show that exposure to diesel exhaust origin secondary organic aerosols may affect higher brain functions such as long-term memory and maternal behavior in a mouse model.

In the atmosphere, semi-volatile organic compounds in diesel exhaust are oxidized and form SOA which is a component of PM_2.5_ [33,34,35]. Currently, SOA formation occurs not only in the atmosphere but also indoors by ozonolysis of terpenoids in home and office spaces [8]. It was also reported that ozone can react with VOCs from laser printers during printing processes to form SOA [9]. The daily PM_2.5_ environmental standard (particles less than 2.5 μm in aerodynamic diameter and respirable fraction of particulate matter) of Japan is less than 35 μg/m^3^. In the present study, there are two possibilities of formation of SOA by oxidative reaction from chemicals on the surface of particles and particle-free volatile products. Primary particles may easily convert to SOA. In our previous studies, we examined the effects of nanoparticle-rich diesel exhaust exposure in mice and we found that moderate doses (approximately 35 μg/m^3^ in the exposure chamber) did not affect the brain functions and high dose-DE (three times above the moderate level in the exposure chamber) significantly affects learning and memory functions in mice [12,14,15]. Thus, we decided to examine the effects of diesel engine exhaust origin SOA at a high-dose level. Our previous and present studies indicate that high doses in our exposure chamber, acute exposure affect brain functions and low doses, chronic exposure may not affect brain functions. A recent interesting study indicated that exposure to SOA from aromatic hydrocarbons in gasoline in the lower 48 states in the USA was associated with premature mortality and social cost due to health impacts [36]. Therefore, from the point of view of environmental health research, the study of the health risk effects of SOA is essential to protect public health.

Regarding *in vivo* studies, it was reported that inhalation of toluene-derived SOA for seven days in ApoE^-^/^-^ mice could cause up-regulation of endothelin-1, heme oxygenase 1 and the appearance of increased matrix metalloproteinase-9, but did not cause inflammatory responses in lung [37]. Moreover, breathing pattern in rats was changed by SOA produced from vehicular emissions and the numbers of total cells, macrophages and neutrophils in the bronchoalveolar lavage and *in vivo* chemiluminesence were significantly increased in their lungs [38]. In a human study, elderly subjects with a history of coronary artery disease showed positive associations of electrocardiographic ST-segment depression with primary combustion aerosol and gases, but not secondary organic aerosols or ozone [39]. Our recent study demonstrated that proinflammatory cytokines, their transcription factor and neurotrophin mRNAs were remarkably increased in lung of mice following a single intranasal instillation of DE-SOA [10]. However, there are limited reports regarding the effects of SOA exposure on higher brain functions such as learning and memory and maternal behavior. This situation prompted us to do the present study.

Our previous studies have shown that exposure to nanoparticle-rich diesel exhaust impaired hippocampus-dependent spatial learning ability and novel object recognition ability accompanied with up-regulation of NMDA receptor expressions in mice [12,14,15]. In the present study, we found that exposure to DE-SOA for one or three months impaired novel object recognition ability accompanied with alteration of NMDA receptor subunit expressions in the hippocampus of mice. Regarding the discrimination index which indicates discriminative activity between new and old objects, it was highly affected by three month exposure to DE-SOA. The NMDA receptors are heteromeric protein complexes that consist of different subunits (NR1_a-h_, NR2A-D, NR3) which possess distinct biophysical properties [40]. The NMDA receptors and their subunits play important roles in synaptic plasticity which is essential for learning and memory functions [41]. The activation of NMDA receptors requires the binding of amino acid neurotransmitter glutamate to NR2 and membrane depolarization and the binding of glycine or d-serine to the glycine site of NR1 [42,43]. In our present study, one month DE-SOA exposure increased NR1 mRNA expression and not affect NR2A and NR2B in the hippocampus of mice. However, after three months of exposure to DE-SOA, increased NR1, but decreased NR2A expressions were observed. Thus, we suggest that duration of exposure may influence the NMDA receptor composition.

It has been reported that in a rat study, cardiac and pulmonary oxidative stress were observed after exposure to SOA including coal-fire power plant emissions [44]. 8OHdG is commonly used as a marker of DNA oxidative stress [27,28] and 8OHdG levels increase in tissue, blood and urine in pathological condition such as cancer, inflammation, diabetes and neurodegeneration. We examined the plasma 8OHdG level in the present study and found that three months of exposure to DE-SOA caused increased plasma 8OHdG levels in DE-SOA-exposed mice. We suggest that oxidative stress produced by DE-SOA may trigger neurotoxicity and which in turn induced the observed learning and memory deficits.

**Figure 5:**
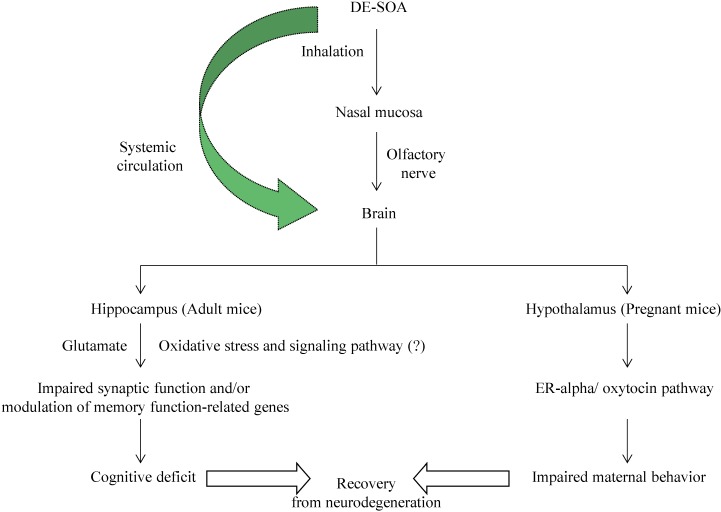
Hypothetical pathway of DE-SOA induced neurotoxicity.

Regarding maternal behavior, our present study showed that nesting and crouching were significantly impaired in dams exposed to DE-SOA at PND5, however, nesting and crouching were recovered at PND12. We found ifferent effects of maternal behavior in DE- and DE-SOA-exposed dams at PND 5 or PND12. It may be due to some recovery from the toxic effects of constituents included in DE-SOA at PND12. The body weight of pups, dams and weight of whole brain and some areas of brain (olfactory bulb, hippocampus and hypothalamus) were not different among the groups. Maternal behaviors during early postnatal period are thought to be important for the normal developmental process of their offspring. The medial preoptic area (MPOA) of the hypothalamus is critical for maternal behavior [45,46,47]. Estrogen sensitivity through its receptor is important for regulation of maternal behavior [48]. In rodents, activation of oxytocin receptor and estrogen receptor in the MPOA are necessary for nest building, pup retrieval, and pup licking/grooming [29,30,49]. In addition, it was suggested that ERα is required for induction of OT receptor by estrogen [50]. Most studies have evaluated the effects of maternal exposure to environmental chemicals or pollutants on the offspring and our present study has demonstrated the effects of exposure to diesel engine derived SOA on maternal behavior in female mice during their lactating period. In the present study, abnormal maternal behaviors (e.g., delay pregnancy, eating pups) before and just after birth were observed. We also found that ER α and OTR mRNAs in the hypothalamus were significantly reduced in female mice exposed to DE-SOA before pregnancy and this was consistent with our immunohistochemical analysis results. Thus, we suggest that DE-SOA may affect maternal behavior through the estrogen-oxytocin receptor pathway in the hypothalamus of mice. It was reported that maternal behavior in mice depends on the detection of odorants and or pheromones released from pups [51,52]. Recent study has indicated that maternal behavior was impaired in female mice lacking adenylyl cyclase which is coupled with odorant receptor in olfactory epithelium [53]. Therefore, another possibility is that DE-SOA may affect odorant receptors in the female mice and they could not detect the odorants and pheromones from pups and then these mice may not show maternal behavior after childbirth. The hypothetical pathway of DE-SOA-induced neurotoxicity is shown in Figure 5.

To the best of our knowledge, this is the first study on the effects of whole body exposure to SOA on higher brain functions such as memory function and maternal behavior using an animal model. We suggest that further study is necessary to elucidate whether there is a similar mechanism in people living in areas of high air pollution or not. Animal experiments showed that inhalation exposure to diesel exhaust during a critical period of brain development such as gestational or lactational period affects sexual differentiation by modulating the expression of estrogen receptors α and β in the mouse cerebrum [54]. Moreover, gestational exposure to low levels of diesel exhaust reduces spontaneous locomotor activity and alters the levels of monoamine neurotransmitters such as dopamine and noradrenaline and their metabolites in different brain regions of young adult mice [55]. Furthermore, epidemiological studies in humans have reported that long-term maternal cigarette smoking during pregnancy affects academic achievement and cognitive abilities in children and young adults [56]. It was suggested that human epidemiological studies and animal experiments show similar patterns of central nervous system damage including oxidative stress and neuroinflammatory processes [57], and our *in vivo* animal model should be helpful for understanding the neurotoxic effects of ultrafine particle-rich DE origin SOA in human.

The characterization, physical and chemical properties of SOA are complex and, better understanding of SOA formation and biological effects could help protect the public from health risks and the effects of climate change. The constituents of SOA in the present study were measured and many kinds of substances were detected. Among them, the major toxic substance candidates are organic hydrocarbons. These organic hydroarbons, especially, water soluble organic hydrocarbons may reach the brain via the systemic circulation or via an olfactory nerve route and may trigger neurotoxicity effects such as learning and memory deficits and impaired maternal behavior in mice. Finally, our present study contributes to the understanding of the health risk effects of diesel engine-generated SOA on higher brain functions. Further studies are needed to explore the potential route of entry of SOA to the brain and its mechanism(s) of action.

## 5. Conclusions

Our present results based on a novel object recognition test and maternal behavioral assessment suggest that the constituents of diesel exhaust may be associated with observed changes in learning performance and maternal behavior in mice. Our present findings also indicate that ultrafine or nano sized particles from environmental pollutants which can change into organic aerosols may affect not only on cardiovascular and pulmonary system, but also the higher functions of the central nervous system.
